# Developing a Protocol-Based Expressive Therapies Continuum Assessment Profile (ETC-AP): Current Achievements and Future Perspectives

**DOI:** 10.3390/bs16050640

**Published:** 2026-04-24

**Authors:** Elza Strazdiņa, Viktorija Perepjolkina, Anda Upmale-Puķīte, Elīna Akmane, Jana Duhovska, Kristīne Mārtinsone

**Affiliations:** Department of Health Psychology and Pedagogy, Rīga Stradiņš University, LV-1007 Riga, Latvia

**Keywords:** Expressive Therapies Continuum, protocol-based assessment profile, criteria-guided observation, activation-inhibition, integration midpoints, trauma-informed assessment, regulation-oriented formulation, normalized profile, interpretation algorithm, case illustration

## Abstract

Art therapy assessment benefits from analytical clarity while preserving non-directive, process-sensitive practice. Although the Expressive Therapies Continuum (ETC) is widely used to conceptualize sensory, affective, cognitive, and symbolic processes in art-making, ETC-informed assessment often relies on implicit clinical reasoning, limiting transparency and interdisciplinary communication. This article presents the developmental stage of a protocol-based Expressive Therapies Continuum Assessment Profile (ETC-AP) developed at Rīga Stradiņš University. The ETC-AP differentiates activation and inhibition patterns around integration midpoints and organizes observation in a defined five-step interpretive sequence without positioning the method as a psychometrically validated test. It combines (i) a uniform three-task, non-directive administration with a brief post-task inquiry; (ii) criteria-guided coding of observable features across three artworks and process notes; and (iii) 0–100 descriptive profile indicators to support within-case pattern description and professional dialogue. An illustrative case vignette shows how the ETC-AP can generate trauma-informed, regulation-oriented hypotheses about channel accessibility and cautious regulation-oriented sequencing, while remaining subordinate to clinical judgment and context. Key boundaries include incomplete operational coverage in some inhibition ranges, limits of static documentation for process-dependent markers, and the need for structured training materials and programmatic studies of reliability, feasibility, and sensitivity to change.

## 1. Introduction

According to the Clinical Guidelines for Art Therapy approved by the National Health Service of Latvia (Medicīniskās tehnoloģijas mākslas terapijā, 2010), art therapists are required to conduct a comprehensive initial assessment of a client’s/patient’s physical, psychological, and social functioning. This typically involves integrating interviews and validated questionnaires with art-based assessment approaches that can access clinically meaningful information through creative expression (Strazdiņa, 2016). Globally, however, art therapy assessment continues to face a persistent methodological tension: clinicians value non-directive, process-sensitive practice, while research and service contexts increasingly require transparency, comparability, and communicability of assessment reasoning (Betts, 2005).

Within this landscape, the Expressive Therapies Continuum (ETC), originally conceptualized by Kagin and Lusebrink (1978) and further developed by Lusebrink (1990, 2010), remains one of the most influential frameworks for understanding how sensory, affective, cognitive, and symbolic processes are expressed and integrated in art-making. Despite its extensive clinical use, ETC has largely remained a descriptive framework. In assessment contexts, ETC-informed formulations are frequently generated through implicit clinical reasoning and narrative interpretation rather than through explicitly structured analytic logic. This limits the transparency of how conclusions are derived and makes professional communication and comparability across clinicians and settings more difficult.

Importantly, this limitation does not lie in ETC theory itself, but in the lack of a clearly articulated operational framework that translates ETC concepts into a defined sequence of observation and interpretation without turning artistic engagement into a test performance or positioning outputs as diagnostic conclusions.

In response to this gap, systematic work was initiated in 2013 at Rīga Stradiņš University (RSU), Latvia, in collaboration with Vija Bergs-Lusebrink. Initial RSU research focused on identifying and refining expressive features and criteria (Meistere-Peltonena, 2016; Dimsone, 2016; Ruttule, 2016; Steinerte, 2021). The current stage advances this work by proposing a regulation-oriented operational approach that makes activation and inhibition patterns explicit across ETC continua—an emphasis that is particularly relevant in trauma-informed practice, where protective inhibition, shutdown, and constrained access to sensory and affective channels are common clinical phenomena (Hinz & Lusebrink, 2024; Haeyen & Wanten, 2024). A growing body of literature suggests that art therapy and related creative arts therapies may be clinically meaningful in work with adults with PTSD and trauma-related difficulties, although the evidence base remains limited and methodologically heterogeneous (Baker et al., 2018; Maddox et al., 2024; Schouten et al., 2014). Reviews indicate potential benefits for trauma-related distress and broader aspects of functioning, while also emphasizing the need for more rigorous and standardized studies (Baker et al., 2018; Wang et al., 2025). In trauma-informed practice, this is particularly relevant where direct verbal processing may be difficult or dysregulating, supporting the broader clinical rationale for structured, art-based assessment approaches (Schouten et al., 2014; Westfall & Nemeroff, 2016).

In Latvian contexts, the above-described line of research and clinical development has been referred to as Mākslinieciskās ekspresijas pakāpenisko līmeņu modelis (MEPL). In the present article, to align with international terminology, we use the term protocol-based Expressive Therapies Continuum Assessment Profile (ETC-AP) to denote the defined administration procedure, the criteria-guided observation approach, and the structured interpretation sequence presented here.

This article presents the current developmental stage of the ETC-AP. The ETC-AP is introduced as a methods-oriented, criteria-guided framework designed to externalize ETC-informed clinical reasoning rather than as a psychometrically validated measurement instrument. While it does not yet meet the standards of a standardized or fully validated assessment method, its primary contribution lies in structuring and making explicit the observational and interpretive processes that are often implicit in ETC-informed practice, thereby enhancing transparency and procedural consistency.

The approach does not provide normative data, diagnostic classification, cut-off scores, or outcome evaluation, and should not be interpreted as a standardized test. Instead, it offers a structured procedure for organizing the observation and interpretation of expressive processes within a single assessment context, with particular relevance for trauma-informed, regulation-oriented formulation. Profile outputs should therefore be understood as descriptive indicators derived from a defined assessment procedure and not as independently verifiable or diagnostic results.

The ETC-AP is not yet a fully established clinical tool and remains at a developmental stage. At present, it is primarily intended for use in research contexts and by trained art therapists familiar with ETC theory, particularly for research and exploratory purposes, rather than for independent routine clinical application.

To address the need for a transparent and operationalized assessment framework, this article explores the following questions: 1. How can the descriptive principles of the ETC be translated into a structured, criteria-guided observation protocol (ETC-AP)? 2. What scoring logic and normalization procedures are required to transform qualitative art-based observations into a visual, comparative profile? 3. How can a multi-step interpretation algorithm support the generation of trauma-informed clinical hypotheses? An illustrative case vignette demonstrates how the ETC-AP can support trauma-informed hypothesis generation and regulation-oriented clinical considerations while remaining subordinate to context, consent, and clinical judgment.

## 2. Theoretical Framework: From Descriptive ETC to an Operational, Regulation-Oriented Framework

### 2.1. The Original ETC Framework

The Expressive Therapies Continuum (ETC) provides an integrative structure for understanding how artistic expression reflects and mediates information processing, emotion regulation, and meaning-making. Grounded in art therapy theory and neuropsychological perspectives, the ETC describes creative functioning across three bipolar levels of increasing complexity: Kinesthetic-Sensory (K-S), Perceptual-Affective (P-A), and Cognitive-Symbolic (Co-Sy) (Lusebrink, 1990, 2010; Hinz, 2019). In clinical work, adaptive functioning is often reflected in flexible access to these levels and the capacity to shift among them in relation to the context and regulatory demands. Conversely, pronounced reliance on one pole (e.g., rigid perceptual structuring) alongside inhibition or constrained access to its counterpart (e.g., restricted affective engagement) may be consistent with difficulties in modulation, integration, or tolerance of experience (Hinz, 2009).

While the ETC is widely used as a descriptive clinical map, assessment applications often require a clearer way to organize observation and communicate patterns without reducing them to diagnostic or test-like claims.

### 2.2. The Refined Operational Model: Axis Representation and Defined Ranges

**Figure 1:**
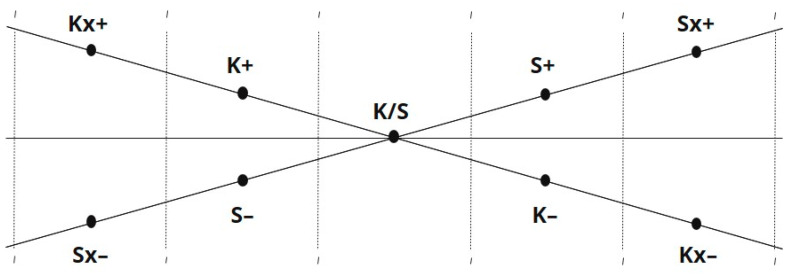
Graphical model of ETC-AP’s Kinesthetic and Sensory Level.

Building on Lusebrink’s original schema, the ETC-AP developed at RSU introduces a refined graphical and operational model to represent the direction and intensity of artistic engagement within ETC continua. While Lusebrink (1990) conceptualized these dimensions as functional continua, the refined model visualizes each continuum as an axis (see Figure 1). As part of the ETC-AP development, a refined terminology is used that differs slightly from the original and commonly used ETC terminology. The newly defined terms, their operational definitions, and the corresponding activation-inhibition ranges are presented in Appendix A, Table A1.

In this graphical representation, the two axes within a level intersect to emphasize their dynamic interdependence. Importantly, the intersection is not treated as a numerical “zero point.” Instead, it represents the integration midpoint (Range of Integration and Balance), denoting a state where expressive qualities of both continua can be used in a relatively balanced and coordinated manner. In clinical terms, this midpoint reflects a potential for the adaptive integration of resources within the level (e.g., K/S, P/A, or Co/Sy) under the current conditions of the assessment context.

To translate this visual model into a shared clinical language, the ETC-AP defines observation ranges that differentiate activation, inhibition, and more extreme regulatory patterns.

### 2.3. Dimensions of Activation and Inhibition

To support consistent clinical coding, each axis is divided into four defined ranges situated symmetrically around the integration midpoint. These ranges operationalize the intensity of the client’s/patient’s engagement with the media and the resulting expressive features:

- Moderate Dominance (+): Indicated by symbols such as K+, S+, etc. This represents high but functional activation, where the continuum’s characteristics are pronounced and constructive.
- Extreme Hyper-arousal (x+): Indicated by Kx+, Sx+, etc. This represents an Extreme Activation, where the continuum dominates the expression to a degree that may become chaotic, rigid, or overwhelming.
- Moderate Inhibition (–): Indicated by K–, S–, etc. This reflects hypo-activity or diminished engagement with the continuum’s specific qualities.
- Extreme Hypo-arousal (x–): Indicated by Kx–, Sx–, etc. This defines the Extreme Inhibition, signifying a complete block or inability to access that particular mode of expression.

This range structure enables the ETC-AP to represent not only directional tendencies in expression but also whether engagement is organized around activation versus inhibition and how these tendencies relate across continua.

### 2.4. Clinical and Methodological Utility of the Refined Model

This refined graphical representation serves three primary functions within the ETC-AP. First, it provides an at-a-glance visualization of a client’s/patient’s expressive regulation profile, supporting the structured formulation and communication of observed patterns. Second, it supports the examination of relationships between opposite continua within each level. On the Kinesthetic-Sensory (K-S) and Perceptual-Affective (P-A) levels, the model can illustrate how pronounced activation in one continuum (e.g., perceptual rigidity, Px+) may co-occur with inhibition or blockage of the opposite continuum (e.g., affective inhibition, Ax–). However, this relationship is not assumed to be strictly reciprocal. Clinical observation (including the illustrative case in this article) indicates that a continuum may be inhibited independently without a corresponding hyperactivation of the opposing continuum.

This principle is also less straightforward at the Cognitive-Symbolic (Co-Sy) level. Unlike the more physiologically proximate lower levels, Co-Sy engagement often reflects higher-order synthesis, and cognitive and symbolic processes may co-occur in complex combinations rather than functioning as simple bipolar opposites.

Third, the model aligns with the ETC-AP’s criteria-guided observation and profile construction. Observed expressive features are mapped onto axes and ranges, supporting a coherent visual profile that helps clinicians and researchers organize qualitative observation into a defined interpretive structure without treating the client’s/patient’s work as a test performance.

### 2.5. Methodological Principles: Criteria-Guided Scoring and Profile Normalization

The ETC-AP uses a criteria-guided approach that moves away from global judgments toward the structured observation of explicitly defined expressive features. Each feature is operationalized through criteria assigned to specific ranges (e.g., Kx+, K+, K/S integration midpoint, etc.).

A key methodological strength of the ETC-AP is its cumulative scoring logic across a uniform sequence of three distinct art-making tasks. While individual criteria are assessed dichotomously (0/1) for each task, the integration of data from all three artworks allows for the emergence of a quasi-continuous scale. This transition from binary observations to aggregated data enables the measurement of expressive density and intensity through a structured numerical framework. At this developmental stage, the resulting values are treated as profile indicators that summarize observed features within the assessment context, rather than as psychometrically calibrated measurements with proven interval or ratio properties.

The ETC-AP profile values are treated as a quasi-continuous descriptive indicator, based on two methodological considerations:

1. Aggregation as an Intensity Proxy: By summing binary criteria across multiple tasks, the ETC-AP generates a score that reflects the frequency and persistence of specific expressive features. A zero score in this system represents a “baseline absence” within the assessment context, signifying that none of the operationalized criteria were met during the session.
2. Standardized Comparability through POMP: To address the varying number of criteria across different continua (or axes), the ETC-AP employs the Percent of Maximum Possible (POMP) transformation (J. Cohen et al., 1999). This converts raw cumulative frequencies into a 0–100 profile range, ensuring that the intervals are mathematically comparable and suitable for quantitative profiling.

However, the inherent asymmetry in the number of criteria per activation range necessitates a proportional weighting logic. To ensure that ranges with a higher density of criteria do not disproportionately skew the overall profile, a weighting factor is applied based on the maximum possible criteria in a given range relative to the total set. This ensures that each range occupies its mathematically “correct” portion of the final profile, providing a transparent and replicable visualization of the client’s/patient’s state on the axis model.

## 3. ETC-AP Development Process

The ETC-AP was developed as a protocol-based assessment profile grounded in ETC theory. Its development follows a theory-driven methodological design, utilizing an iterative process of concept-driven feature generation, criteria sifting, expert review, and applied testing in clinical and educational contexts. Rather than following a psychometric “instrument construction” pathway aimed at establishing a standardized test, the development work focused on (a) making ETC-relevant observations explicit and criteria-guided, (b) ensuring procedural consistency of the assessment setting, and (c) improving the clarity and reproducibility of scoring decisions across raters over time (Anastasi, 1985). At this stage, the emphasis is on establishing procedural consistency rather than psychometric normalization.

The development of ETC-AP can be summarized in three phases.

### 3.1. Phase 1: Conceptualization and Initial Feature Generation (2013–2016, 2021)

The initial work at Rīga Stradiņš University focused on translating ETC theory into a structured observation and coding system. This development drew on foundational ETC theory and established art-based assessment approaches to inform early feature generation and operational definitions (B. M. Cohen et al., 1988; Gantt & Tabone, 1998; Hays & Lyons, 1981; Lusebrink, 1990). The aim in this phase was to articulate ETC-consistent patterns of preferred use, overuse, and underuse as observable markers applicable to spontaneous art-making (Lusebrink, 1990). A structured three-task format was also introduced to support procedural consistency while preserving the non-directive ethos of art therapy (Betts, 2005).

### 3.2. Phase 2: Operational Refinement and Criteria Sifting (2017–2024)

As pilot applications and early research use progressed, development efforts focused on refining criteria definitions and improving scoring consistency (Skara, 2017; Danilāne, 2017). This included iterative revision of feature descriptions based on applied use and rater discussion, with attention to clarity, observability, and consistency. While this process resulted in a more stable and operationalized set of criteria, some activation and inhibition ranges remain under-defined at the present stage, reflecting current limits of formalized observation rather than the absence of clinical phenomena.

### 3.3. Phase 3: Development of Profile Construction Logic (2025–2026)

The most recent phase focused on strengthening how observed criteria are aggregated and represented as an ETC-AP profile (see Section 2.5 and Section 5.1). This included the introduction of normalization procedures to support comparability across continua and improve interpretability of results (J. Cohen et al., 1999). At the current developmental stage, these outputs are treated as structured, normalized indicators for within-case description and interpretation, rather than as psychometrically validated measurements.

## 4. Structured Assessment Procedure (ETC-AP)

The ETC-AP is designed as a defined, single-session (90 min), uniform, structured assessment procedure that remains non-directive in the art-making itself. It is intended for use across clinical, rehabilitation, and educational contexts, combining a consistent administration structure with attention to the client’s/patient’s own meanings and pacing. This approach aims to support the transparency and comparability of observations while preserving clinical sensitivity and core art therapy principles (Betts, 2005; Martinsone, 2011).

### 4.1. Session Plan and Uniform Instructions (ETC-AP Administration)

#### 4.1.1. Material Setup and Uniform Environment

**Figure A1:**
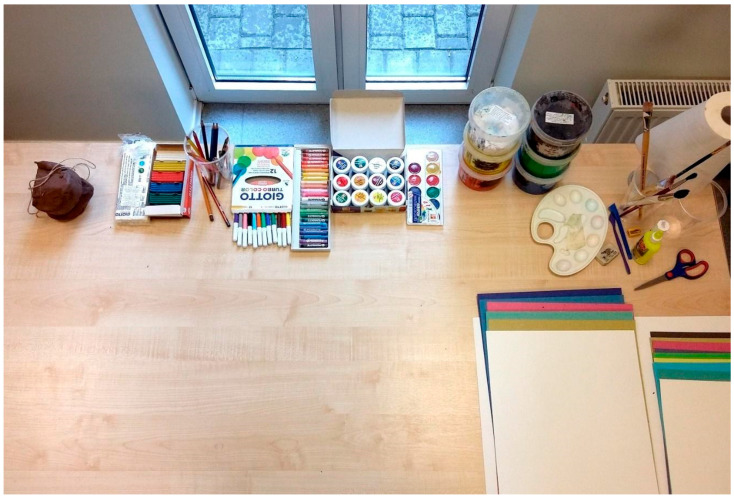
Spatial arrangement of materials.

Before the client’s/patient’s arrival, the art therapist prepares the workspace using a defined spatial arrangement of materials to ensure comparable access across sessions. Drawing and painting media (dry and fluid) are placed directly in front of the client/patient, while auxiliary tools (e.g., brushes, water container), white paper in several sizes, and colored paper are positioned on the right side of the table. The same material set and arrangement are used across administrations to reduce contextual variability and support the within-procedure comparability of observation in the ETC-AP framework. A detailed inventory of the art materials and auxiliary tools, as well as a figure depicting the material layout, is provided in Appendix A.2 (Table A2 and Figure A1).

#### 4.1.2. Session Phases and Timing

The session follows four phases:

Preparatory phase: the therapist completes the material setup described above.

Initial phase (up to 5 min.): the therapist explains the session structure and provides the uniform instructions.

Working phase (up to 75 min.): three repeated cycles, each consisting of

(a) art making (up to 15 min.), followed by
(b) a brief post-task inquiry (up to 10 min.).

During the inquiry, the therapist and client/patient may adjust seating to support verbal exchange.

Closing phase (up to 10 min.): a brief concluding conversation supporting the integration of the session experience and checking the client’s/patient’s state before ending.

#### 4.1.3. Three-Task Structure and Verbatim Instruction

The working phase comprises three tasks that are identical in structure and differ only in the client’s/patient’s spontaneous engagement with materials. This repetition supports the cumulative observation of consistency, variability, and shifts in activation, inhibition, and integration across the session.

Before Task 1, the therapist informs the client/patient that three artworks will be created and that a short conversation will follow each one. The therapist briefly names the available materials and invites the client/patient to explore them if desired. The core instruction is delivered verbatim and repeated before each artwork to maintain procedural consistency:

“You are invited to create an artwork using any of the materials provided. There is no right or wrong way to do this. You can choose how you work and when you feel the artwork is finished.”

Approximately five minutes before the end of each art-making period, the therapist provides a neutral time cue (e.g., “We have about five minutes left”) to support pacing without directing content.

#### 4.1.4. Post-Task Inquiry: Core Prompts and Trauma-Informed Boundaries

After each artwork, a brief semi-structured inquiry (up to 10 min) supports the observation of processes relevant to the Cognitive-Symbolic level (Co-Sy) and integration. The inquiry is conducted in a consent-based, trauma-informed manner: it does not require the disclosure of traumatic content, and participants may decline any question without consequence. If the client/patient chooses not to verbalize, this is documented as contextual information and is not interpreted as a deficit.

Two core prompts are always considered:

1. “Tell me about your artwork—what is shown here?”

2. “What is this, and what did you mean by it?”

Depending on the client’s/patient’s capacity and tolerance, additional neutral prompts may be used to clarify meaning-making and the relational organization of image elements, for example:

3. “What does this mean to you?”

4. “Is there any relationship between these elements?”

5. “Do you have any associations connected with this—does it relate to something in your life, and if so, how?”

Prompts may be adjusted to the client’s/patient’s developmental level and clinical condition (e.g., simplified language and fewer prompts when needed), while preserving the same inquiry logic and non-directive stance.

### 4.2. Documentation and Procedural Fidelity

Across the three task cycles, the therapist documents: (a) materials used, (b) process observations (e.g., pacing, initiation, and shifts across tasks), (c) product characteristics, and (d) verbal reflections where provided. By combining a defined material setup, a uniform three-task structure, consistent instructions, and a core post-task inquiry, the ETC-AP supports procedural consistency and transparent, criteria-guided documentation while keeping content generation fully client/patient-led.

The post-session evaluation is conducted using the ETC-AP Feature Rating Table (see Appendix B). Each of the 23 features is evaluated based on its specific criteria.

1. Coding: The rater determines the presence (1) or absence (0) of each criterion for each of the three artworks.
2. Aggregation: Codes are aggregated across the three tasks to summarize how consistently specific criteria appear within the assessment context, yielding cumulative frequencies at the feature/range level.
3. Profile Construction: Aggregated values are converted to a common 0–100 normalized profile using Percent of Maximum Possible (POMP) transformation, with proportional weighting applied to reduce the visual imbalance arising from unequal criterion density across ranges (see Section 2.5). At this developmental stage, profile outputs are treated as descriptive indicators to support within-case pattern description and professional communication, not as psychometrically calibrated measurements.

## 5. Data Processing and Interpretation

### 5.1. Data Processing Algorithm

The transition from qualitative observations to a uniform, criteria-guided profile representation is conducted through a multi-step data-processing procedure. The processing follows sequential steps, with each stage implemented in the Excel-based scoring file (see Supplemental Material S1). The presented data-processing algorithm should be understood as a methodological proposal designed to support transparent profile construction. It has not yet undergone formal psychometric evaluation.

Step 1: Identifying Baseline Parameters (Columns B and C in the scoring file)

Before data entry, the scoring file specifies the calculation parameters for each activation range:

- Column B (Criteria Count): This represents the number of unique criteria defined for a specific expressive feature within a range.
- Column C (Maximum Possible Score (*MS*)): Since the ETC-AP utilizes a three-task structure, the maximum number of points for any range is the number of criteria multiplied by three.^1^ This reflects the cumulative nature of the assessment across the entire session.

Step 2: Raw Data Entry (Column E)

After reviewing all three artworks (and the observed process), the rater enters the cumulative count of observed criteria into the Client’s/Patient’s Initial Score - Column (E). The value in E can range from 0 to the value specified in Column C.

Step 3: Determining the Weighting Factor (Column D)

To compensate for the unequal distribution of criteria (e.g., Kx+ having 21 possible points while K/S has only 3), a weighting factor (*W*) is applied. It is calculated as:

(1) $$W_{i}\;=\;\frac{C_{max}}{C_{i}},$$

where *C_max_* is the global constant for the level, and *C_i_* is the maximum points for the specific range. This supports balanced representation across ranges with different numbers of defined criteria, so that ranges with fewer criteria are not systematically underrepresented in the profile.

Step 4: Calculating POMP and Weighted Value (Columns F and G)

- Column F (POMP): Calculates the percentage of the maximum possible score (POMP) for that specific range:

(2) $${POMP}_{i}=\left(\frac{{RS}_{i}}{{MS}_{i}}\right)\times 100,$$

where *RS_i_* (Raw Score) represents the Client’s/Patient’s Initial Score (Column E), which is the cumulative count of observed criteria across the three assessment tasks for the specific activation range, and *MS_i_* (Maximum Score) represents the Maximum Possible Score (Column C) for a specific activation range.

Column G (Weighted Value *S*): After calculating the density of expression within a specific range (POMP), the ETC-AP applies a weighting factor to support consistent profile interpretation across the axis model. This step transforms the percentage into a Weighted Value (*S*), which accounts for the varying number of criteria per range. The formula for the Weighted Value (*S*) is as follows:

(3) $$S_{i}\;={POMP}_{i}\times W_{i},$$

where *S*_i_ (Weighted Value): the final score (Column G) used to map the client’s/patient’s expressive profile on the axis model; POMP: the standardized density score (Column F) calculated in the previous step, representing the client’s/patient’s observed profile intensity relative to the maximum possible score; *W_i_* (Weighting Factor): the coefficient (Column D) calculated at the third step. This factor equalizes the impact of each activation range, supporting more interpretable profiles when comparing ranges with fewer criteria to ranges with more criteria.

Step 5: Normalization (Column H) The final stage of the data processing algorithm is the calculation of the Normalized Score (Column H). This step is essential for transforming the Weighted Values (*S*) into a common 0–100 metric for each ETC continuum. Normalization eliminates the scale variations introduced during the weighting process and enables the creation of a clear profile representation on the axis model, with each continuum expressed relative to its maximum possible value under the defined criteria.

The Normalized Score is calculated using the following formula:

(4) $${Norm\%}_{i}\;=\;\left(\frac{S_{i}}{{Smax}_{i}}\right)\times 100,$$

where *Norm%* (Normalized Score): The final percentage (Column H) representing the proportion and intensity of a specific activation range for a specific continuum (e.g., Kx+ or K/S) within the overall profile; *S_i_* (Weighted Value): The specific weighted value calculated for that activation range of the specific continuum (Column G); *Smax*_i_ (Maximum Weighted Value): The theoretically highest possible weighted value for that specific activation range of the continuum. This value is achieved if the client/patient obtains the maximum raw score across all three tasks (i.e., when POMP = 100).

This normalization allows the art therapist to immediately identify dominant activation or areas of significant inhibition within the client’s/patient’s artistic expression. It provides a shared, defined frame of reference for comparing continua (e.g., Affective vs. Cognitive values) within the same profile representation.

At this developmental stage, these values should be understood as normalized profile indicators intended to support transparent description and interpretation, rather than as psychometrically calibrated measurements.

#### 5.1.1. Methodological Logic of Profile Indicators

The percentage values generated by the algorithm (ranging from 0% to 100%) represent the relative density of specific operationalized criteria identified within the scoring framework, rather than an ontological presence or absence of an ETC component in the client’s/patient’s experience. A score of 0% indicates that no predefined markers were observed during the assessment protocol, while a score of 100% signifies that the observed expressive features exclusively met the criteria for that specific range within the normalized scoring procedure. Intermediate percentages (e.g., 15%, 50%, 75%) are common and reflect the cumulative frequency and distribution of identified criteria across the three tasks, allowing for a nuanced representation of expressive activation.

#### 5.1.2. Practical Interpretation of Profile Percentages

To support the practical interpretation of the resulting profile, the following logic should be applied:

∗ Higher Percentages (70–100%): Indicate a high concentration or “density” of observed expressive criteria within a specific range. This suggests that the client/patient consistently utilized expressive features associated with that particular ETC activation or inhibition mode across the assessment tasks.
∗ Moderate Percentages (30–60%): Reflect a moderate presence of scorable markers, indicating that while the expressive mode is accessible, it is not the dominant organizing force in the client’s/patient’s current artistic engagement.
∗ Lower Percentages (1–20%): Suggest that only isolated criteria were identified, reflecting a lower availability or less frequent use of that expressive channel under current conditions.
∗ Zero Percent (0%): Indicates that no scorable criteria from that range were identified under the current protocol. As emphasized in Section 7.3, this should be interpreted as a “baseline absence” within the assessment context rather than an absolute phenomenological absence of that expressive modality in the client’s/patient’s creative process.

### 5.2. The ETC-AP Interpretation Algorithm

To support the clinically grounded interpretation of the ETC-AP, the clinician applies a defined five-step interpretation algorithm. The sequence integrates horizontal (within-level), vertical (within-continuum), and cross-level (integration) analyses to organize normalized profile values into clinically meaningful hypotheses. The algorithm structures how observations are organized and discussed; it does not generate diagnoses or prescriptive conclusions. Throughout, observed patterns are interpreted as state-dependent regulatory organization and cross-checked against process observation and contextual information.

Step I: Relative Continuum Dominance and Inhibition (Horizontal Analysis)

The first step identifies, within each ETC level, which continuum shows relatively higher activation and which continuum shows relatively higher inhibition (or lower availability) under the current assessment conditions. If a range currently has no operationalized criteria, it is coded as N/A and visually greyed; it is treated as not scorable in the current version and is not interpreted.

Key question: Which continuum is most activated at each level, and which appears relatively inhibited/less accessible?

Clinical relevance: This step may indicate the client’s/patient’s primary mode of engagement and potential areas of reduced access. In trauma-informed contexts, such patterns may be discussed as reflecting regulation-related tendencies (e.g., protective inhibition or reduced availability of a channel) rather than deficits.

Step II: Polarity and Energetic Focus (Internal Conflict)

The second step examines polarity within each continuum by assessing whether higher values cluster in activation ranges (+/x+) versus inhibition ranges (–x–), and whether activation and inhibition markers co-occur within the same continuum (e.g., elevated K+ alongside elevated Kx–).

Key question: Is intensity primarily expressed through activation (+/x+) or inhibition (–/x–), and is there evidence of concurrent elevation in the continuum?

Clinical relevance: This step supports state-level interpretation within the ETC framework:

Hyper-arousal: relative predominance of x+ and + ranges.

Hypo-arousal: relative predominance of – and x– ranges.

Step III: Intra-level Midpoint Asymmetry (Vertical Analysis)

This step evaluates within-level asymmetries by comparing integration midpoint values in both directions at the same ETC level (e.g., P/A vs. A/P). The ETC-AP scoring architecture treats each of the three ETC levels (Kinesthetic-Sensory, Perceptual-Affective, and Cognitive-Symbolic) as a functional intersection of two distinct axes, representing the level’s underlying components. While these components (e.g., Perceptual and Affective) form a single continuum at their level, the algorithm identifies and calculates scores for each axis independently, based on specific criteria defined for each activation-inhibition range. Consequently, the profile generates two directional integration indicators for each level (e.g., P/A and A/P; K/S and S/K; Co/Sy and Sy/Co). These should not be interpreted as identical mathematical midpoints, but as indicators of differently weighted integration, reflecting which component serves as the primary organizing force in the expressive process under the current assessment conditions.

Key question: How do the integration midpoint scores differ within the same level?

Clinical relevance: Marked asymmetries may reflect uneven integrative organization—for instance, stronger perceptual organization alongside reduced affective access, which can be read as a containment-oriented regulatory strategy under current conditions. These interpretations remain hypothesis-generating and should be cross-checked against process observations, interview material, and contextual factors (e.g., fatigue, medication effects, pain, psychomotor slowing, and familiarity with materials).

Step IV: Creative-Axis Permeability Across Integration Midpoints (Integrative Capacity)

The fourth step examines the pattern of values across the three integration midpoints (K/S → P/A → Co/Sy) to assess how readily engagement appears to move across levels within the assessment context.

Key question: Is activation evident across the integration midpoints (K/S → P/A → Co/Sy)?

Clinical relevance: Lower midpoint values may suggest constrained integrative throughput under the current conditions of safety and arousal—i.e., limited linkage between sensory grounding, perceptual-affective organization, and cognitive-symbolic meaning-making during the session. Importantly, midpoint values should be interpreted in light of operational coverage: where criteria are not yet defined for specific ranges, low values reflect the current boundary of formalized observation rather than the definitive absence of integrative capacity.

Step V: Therapeutic Strategy and Resource Bridging (“Bottom-Up”)

The final step derives regulation-oriented clinical implications by identifying relatively accessible resources as potential safe entry points and tentative “bridges” toward less accessible channels (e.g., from Perceptual engagement toward Affective or Sensory engagement).

Core principle: When pronounced inhibitory patterns suggest a freeze-aligned or shutdown state, clinicians should avoid directly targeting strongly inhibited ranges. Instead, engagement should prioritize safety, consent, and tolerance.

Action: The relatively dominant or accessible continuum can be used as an initial anchoring pathway to support arousal modulation and to cautiously explore pathways toward less accessible channels, with the aim of supporting an integrative linkage across levels. Any clinical implications remain individualized and must be integrated with interview material, clinical history, and professional judgment.

## 6. Case Study: Clinical Application of the ETC-AP

The following vignette is presented for methodological illustration to demonstrate how the ETC-AP structures observation and makes interpretive reasoning explicit through the five-step sequence (Steps I–V). The ETC-AP does not generate automatic conclusions and is not intended to provide diagnostic statements, symptom measurement, or evidence of treatment efficacy. It also does not directly assess trauma-related conditions; rather, it supports the identification of regulation-related expressive patterns (e.g., activation, inhibition, and integration across ETC levels) that may be clinically relevant within trauma-informed formulation. Accordingly, the clinical implications described in Step V are presented as regulation-oriented hypotheses about potential entry points and bridging considerations, to be integrated with interview material, clinical history, and professional judgment.

### 6.1. Case Vignette: “Kate”

This case example illustrates the application of the ETC-AP interpretation steps (I–V) in a psychiatric rehabilitation context. The participant provided informed consent for the use of clinical material and artwork. All identifying information has been anonymized to protect confidentiality. Identifying details were removed or generalized to reduce re-identification risk. Interpretations are presented as context-dependent hypotheses and are not diagnostic conclusions.

It should be noted that the ETC-AP emphasizes procedural clarity, non-directiveness, and pacing to minimize the risk of distress or re-traumatization. Kate was informed in advance about the session structure (three art-making periods, each followed by a brief optional inquiry), and was explicitly told that she may pause, modify, or decline any part of the procedure at any time without consequences. The art-making tasks do not require the disclosure of traumatic content. During and after the session, pacing and stabilization were prioritized, and a brief debriefing check-in confirmed tolerability and current safety before Kate returned to routine care.

Clinical presentation. Kate, a 19-year-old woman, was admitted to a psychiatric rehabilitation unit following a high-lethality suicide attempt. The admission occurred within a pattern of repeated hospitalizations over a six-month period. Presenting concerns included severe depressive episodes, generalized anxiety, and trauma-related symptoms consistent with post-traumatic stress disorder (PTSD).

Psychosocial background. Kate’s developmental history includes chronic adverse childhood experiences. She grew up in a family context characterized by parental alcohol dependence and recurrent interpersonal violence, and she reported prolonged emotional abuse. Such conditions are commonly associated with heightened threat monitoring and reduced experiences of psychological safety.

Coping and defensive functioning. Kate presented with a “high-functioning” profile in academic settings, maintaining strong performance and outward stability. She described this strategy as effortful and associated with increased internal anxiety. She reported prior alcohol use as a maladaptive coping strategy, discontinued by the time of assessment. Despite having social contacts, she described progressive withdrawal and emotional constriction.

### 6.2. Case Analysis: Application of the ETC-AP Interpretation Algorithm

**Figure 2:**
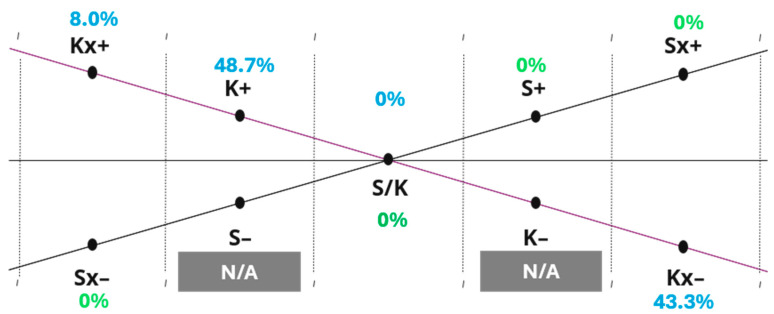
The ETC-AP: Kinesthetic-Sensory (K-S) level. Normalized profile values mapped onto the K-S axes. Grey shading denotes ranges coded as N/A (not scorable in the current ETC-AP version due to a lack of operationalized criteria).

**Figure 3:**
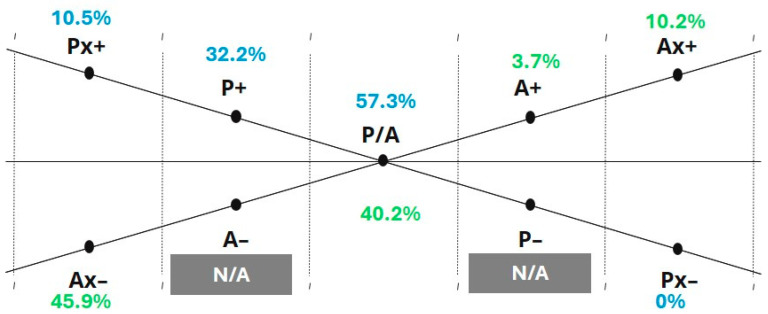
The ETC-AP: Perceptual-Affective (P-A) level. Normalized profile values on the P-A axes. The P/A integration midpoint is elevated. Grey shading denotes ranges coded as N/A (not scorable in the current ETC-AP version due to a lack of operationalized criteria).

**Figure 4:**
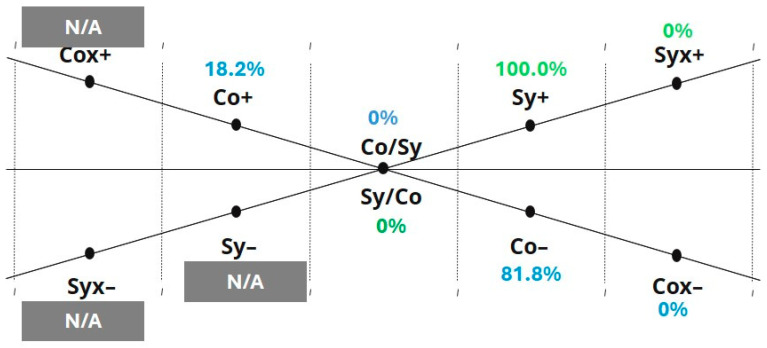
The ETC-AP: Cognitive-Symbolic (Co-Sy) level. Normalized profile values on the Co-Sy axes. Sy+ is elevated (100%), with a relatively prominent Co– range. Grey shading denotes ranges coded as N/A (not scorable in the current ETC-AP version due to a lack of operationalized criteria).

Kate’s ETC-AP results are summarized in Figure 2, Figure 3 and Figure 4 (K-S, P-A, and Co-Sy levels). The five-step sequence below illustrates how the ETC-AP supports structured interpretation by making observation targets and interpretive moves explicit. To support interpretation, the ETC-AP profile should be read as a structured descriptive summary of observed expressive tendencies within the assessment context. The resulting percentages do not represent diagnostic scores; rather, they indicate the relative density of operationalized criteria within a given range across the three tasks. Importantly, the resulting profile and interpretations are presented as trauma-informed, clinically grounded hypotheses that must be integrated with contextual information and process observations rather than treated as diagnostic conclusions or automatic clinical decisions. All three of Kate’s artworks and her reflective statements from the assessment session are provided in Appendix C.

Within this case analysis, all three of Kate’s artworks were evaluated by two raters—one expert and one trained in the use of the ETC-AP. The raters initially conducted independent assessments, after which the results were compared within the ETC-AP working group. Final profile values were established through a consensus-based discussion, including resolution of a small number of discrepancies.

Step I: Relative Continuum Dominance and Inhibition (Horizontal Analysis)

(1) Observations. Marked asymmetries are evident across ETC levels.

K-S level: The Kinesthetic (K) continuum shows relatively higher activation compared to Sensory, while Sensory (S) shows no observable criteria (S range scores = 0% in the present profile).

P-A level: A pattern of relatively stronger Perceptual structuring is evident (e.g., elevated P/A integration midpoint and P+), alongside elevated Affective inhibition markers (e.g., Ax–), suggesting a profile in which perceptual organization is more accessible than affective engagement under current conditions.

Co-Sy level: Symbolic activation is pronounced (Sy+ = 100%), alongside comparatively lower Cognitive (Co) organization in relation to Symbolic engagement, indicating that symbolic immersion is dominant in this profile.

(2) Interpretive considerations (clinical significance).

In ETC terms, strong kinesthetic emphasis can reduce awareness of the sensory component, while sensory emphasis typically slows down kinesthetic action because attention shifts toward sensation (Lusebrink et al., 2013). Within this logic, a K–dominant profile alongside S = 0% can be framed as a state in which action/mobilization is more available than sensory registration or sensory awareness under current conditions.

At the P-A level, a relatively stronger P/A integration midpoint and P+ activation alongside Ax– markers suggest a configuration in which perceptual organization and structure are more available than affective expression under current conditions. In ETC-informed formulation, such patterns can be read as a possible regulatory strategy in which structure, predictability, and controlled perceptual engagement support tolerable functioning when affective access is constrained (Hinz, 2009; Lusebrink, 1990). From a trauma-informed perspective, constrained affective expression may be clinically relevant because posttraumatic stress presentations commonly involve patterns of avoidance and negative alterations in mood/affect, including emotional numbing and reduced access to positive affect, alongside state-dependent shifts in arousal regulation (e.g., oscillation between mobilization and shutdown/dissociative responding) (Biggs et al., 2025; Fonzo, 2018). Accordingly, the present pattern can be treated as a cautious hypothesis that perceptual organization may serve as a relatively safe stabilizing channel for pacing and safety, while Ax– may reflect limited affective availability or protective inhibition under current conditions.

At the Co-Sy level, maximal Symbolic activation (Sy+) is noteworthy because symbolic production can occur without corresponding integrative throughput across levels. ETC theory emphasizes that higher-level symbolic engagement does not automatically imply integration; clinically meaningful change depends on the capacity to link symbolic material with sensory grounding and cognitive organization (Lusebrink, 1990; Hinz, 2009). In trauma-informed contexts, pronounced symbolic immersion may therefore represent a highly accessible expressive channel, yet it still warrants checking whether symbolic content is accompanied by integration midpoints that indicate connection across levels, and whether symbolic engagement functions as expansion, containment, or avoidance within the specific relational and situational context of the session.

In Kate’s case, dominance of the Kinesthetic continuum was reflected in a clear emphasis on action and rhythmic movement during art-making, characterized by medium-amplitude gestures and sustained engagement with the process of movement itself. Notably, she did not engage in exploratory interaction with the sensory qualities of the materials, such as texture, surface, or tactile variation. This pattern may partly reflect familiarity with art-making and an established preference for process-oriented expression. At the same time, from an ETC-informed and trauma-informed perspective, limited sensory engagement may also indicate restricted access to sensory processing. Traumatic experiences involve not only cognitive but also deeply sensory components, and sensory processing channels may become inhibited or avoided when sensory immersion is experienced as overwhelming or threatening (Hinz, 2009). Accordingly, the absence of sensory activation in the present profile may reflect protective inhibition rather than a lack of expressive capacity.

At the Perceptual-Affective level, Perceptual activation was evident through the use of defined forms, figures, and attention to structural detail, with relatively less emphasis on color blending or affective modulation through chromatic exploration.

At the Cognitive-Symbolic level, cognitive organization was reflected in the planning of the artwork and conceptual coherence across the visual compositions. Kate’s behavior, artistic process, and verbal communication were characterized by structure, logic, and order, with relatively restrained affective expressivity. At the same time, her verbal explanations of Artwork 1 and Artwork 2 placed strong emphasis on symbolic meaning, with the artworks explicitly described as representations of emotional states.

Step II: Polarity and Energetic Focus (Internal Conflict)

(1) Observations. At the K-S level, polarity is elevated on both ends of the Kinesthetic axis, with K+ (48.7%) and Kx– (43.3%) showing near-equivalent prominence. This configuration suggests an internal tension on the Kinesthetic continuum, where expressive mobilization and strong inhibitory markers co-occur. In contrast, no comparable conflict is evident on the P continuum: Perceptual energy is more consistently oriented toward activation (P+) rather than split between activation and inhibition. At the P-A level, the overall energetic focus appears asymmetric across continua: on the Perceptual side, emphasis shifts from the integration midpoint toward activation (P+), whereas on the Affective side, emphasis shifts from the midpoint toward inhibition, with Ax– (45.9%) emerging as the most prominent affect-related marker. At the Co-Sy level, symbolic activation is maximal (Sy+ = 100%). On the Cognitive axis, both Co+ and Co– are present, suggesting concurrent cognitive structuring alongside cognitive inhibition rather than a single-direction dominance.

(2) Clinical significance. The near-equivalence of K+ and Kx– may be interpreted as a regulation pattern in which goal-directed activation (mobilization/action) coexists with strong inhibitory forces that constrain or rapidly downshift engagement—potentially consistent with the oscillation between activation and freeze-aligned inhibition under current conditions. In Kate’s case, the presence of the Kinesthetic continuum was observable across all three artworks within the ETC-AP assessment, indicating the consistent accessibility of action-based engagement. K+ activation was reflected in medium-amplitude body movements and sustained energetic investment in the art-making process, particularly evident in Artwork 3, where rhythmic movement with medium intensity was observed. In contrast, Kx– markers were primarily evident in Artwork 1 and Artwork 2, where movement was characterized by irregular rhythmic continuity. This fluctuation between disrupted and more organized rhythmic engagement suggests that mobilization capacity was present but not consistently stabilized across tasks. Within ETC-informed and trauma-informed frameworks, such oscillation may reflect dynamic regulation between activation and inhibition, potentially consistent with alternating states of mobilization and inhibitory down-regulation under current conditions.

Elevated Ax– can be read as a hypothesis of affective containment, where emotional modulation is achieved primarily through inhibition (e.g., suppression/numbing) rather than outward expression; importantly, this should be framed as a trauma-informed, context-dependent hypothesis rather than a trait conclusion. In Kate’s artwork, Ax– was reflected in the absence of color blending and limited chromatic modulation in Artwork 2 and Artwork 3. This was further supported by Kate’s selection of predominantly resistive art materials, such as markers and crayons. Additionally, Kate’s verbal reflection on her artwork was characterized by cognitively mediated descriptions of emotion (e.g., “this reflects how I feel”), indicating the symbolic and cognitive representation of emotional experience rather than direct affective immersion.

The asymmetric energetic focus across P and A—more available perceptual activation (P+) alongside prominent affective inhibition (Ax–)—may suggest that structure and perceptual organization function as a relatively accessible stabilizing channel when affective access is constrained. In Kate’s case, elevated P+ activation was evident in the proportional organization of forms, structured figure construction, and focused attention to visual detail, particularly in Artwork 1 and Artwork 2.

Finally, the Co-Sy level pattern can be interpreted as symbolic immersion co-occurring with cognitive push-pull dynamics—i.e., moments of meaning-making and organization alongside moments of inhibition. In Kate’s work, coactivation was reflected in active planning, structured execution, and coherent integration of artistic elements, as well as clear and logically organized verbal reflection. At the same time, symbolic activation was evident in the personalization and exploration of symbols within the artworks, with symbolic elements explicitly used to represent emotional experiences.

Step III: Intra-level Midpoint Asymmetry (Vertical Analysis)

(1) Observations: A notable discrepancy is present at the Perceptual-Affective integration midpoints, with P/A (57.3%) exceeding A/P (40.2%).

(2) Clinical significance: This asymmetry suggests that, under the current assessment conditions, perceptual organization and structure may be more accessible than affective expression and may function as a regulatory channel that supports containment, predictability, and tolerable engagement when affective access is constrained. This pattern is also observable in Kate’s artworks and creative process, where emphasis was placed on forms and structural details, with comparatively less focus on the exploration and modulation of color. As mentioned before, in trauma-exposed presentations, constrained affective access and/or high effort toward affect modulation (e.g., emotional constriction, numbing, avoidance) are common clinical phenomena and may co-occur with compensatory reliance on controlled, structured modes of engagement.

Step IV: Creative-Axis Permeability Across Integration Midpoints (Integrative Capacity)

This step is not evaluated in the present case analysis due to the currently insufficient number of operationalized integration midpoint features and criteria within the ETC-AP protocol.

Step V: Clinical Implications and Regulation-Oriented Sequencing (“Bottom-Up” Bridging)

Based on the ETC-AP, the following clinical implications may support regulation-oriented sequencing and hypothesis generation regarding safe entry points for engagement. These implications are intended to inform clinical reasoning rather than to prescribe intervention.

(1) Cognitive scaffolding of symbolic material (Co-Sy bridging).

When symbolic activation is high (Sy+), introducing an external cognitive structure (e.g., naming key elements, establishing temporal sequence, narrative organization, “image-to-word” processing) may support bridging across the Co-Sy interface and promote more organized meaning-making. This may be especially useful when cognitive modulation appears variable (e.g., concurrent Co+ and Co– indicators), as a gentle structure can help anchor symbolic material without forcing premature emotional activation.

(2) Perceptual containment and predictability (supporting paced affect access).

Given the relatively strong P/A integration midpoint (P/A = 57.3%), structured perceptual tasks (e.g., framing/bordering, sorting, sequencing) may provide a predictable container that supports paced contact with inhibited affect (Ax–) while maintaining safety, choice, and tolerability. In this formulation, perceptual organization is treated as a potentially accessible stabilizing channel rather than as avoidance per se.

(3) Sensory grounding (supporting tolerable bodily anchoring).

Given S = 0% in the present administration, considering introducing materials with strong tactile and proprioceptive feedback (e.g., clay, textured media) to support gradual re-engagement with sensory experience and strengthen tolerable bodily anchoring may be useful. This may be particularly relevant when inhibitory markers (e.g., Kx–) are prominent.

Importantly, the interpretations generated through the ETC-AP and five-step sequence should not be construed as diagnostic statements, nor should the resulting scores be treated as prescriptive or directly translatable treatment plans. The algorithm is intended to structure and externalize clinical reasoning and to support transparent, communicable, trauma-informed hypotheses regarding regulatory organization; however, these hypotheses require contextual integration with interview material, clinical history, and professional judgment.

In addition, alternative explanations of the observed profile should be considered. For example, patterns such as low activation in specific ranges, elevated inhibitory markers, or pronounced preferences for particular modes of engagement may also be influenced by physical and situational factors (e.g., fatigue, pain, psychomotor slowing, medication effects), task-related conditions, or idiosyncratic artistic habits, including prior familiarity with the materials, habitual material use, and differences in perceived safety or comfort with tactile versus symbolic processes. These considerations underscore that the ETC-AP provides a structured basis for interpretation rather than definitive conclusions, and that any clinical implications must remain individualized and responsive to the client’s/patient’s consent, safety, and the broader context.

## 7. Discussion

### 7.1. Principal Contribution: From Implicit ETC Use to Explicit Operational Reasoning

This article contributes a clearly articulated operational approach to ETC-based assessment by making the underlying logic of observation and interpretation explicit. In many clinical contexts, the ETC is used as a descriptive and integrative map of expressive processes, but assessment applications often remain dependent on implicit clinical reasoning. The ETC-AP addresses this gap by offering (a) defined observation targets anchored in ETC continua, (b) an explicit differentiation between activation, inhibition, and integration as regulation-related phenomena, and (c) a structured five-step interpretation sequence that externalizes clinical reasoning. The central value of the proposed approach is therefore methodological and communicative: it clarifies how ETC-informed assessment reasoning is generated and shared, rather than claiming measurement validity, diagnostic precision, or treatment efficacy.

Interpretive outputs produced through the ETC-AP are formulated as clinically grounded hypotheses rather than diagnostic conclusions or symptom indices. The five-step sequence structures the order of reasoning—what is attended to first, how patterns are organized, and how regulation-oriented implications are derived—yet it does not replace clinical judgment. The approach supports transparency by making interpretive moves visible to other professionals and by clarifying which features of artistic engagement are treated as meaningful within this model. This is particularly relevant in trauma-informed contexts, where overly definitive interpretation may inadvertently replicate coercive dynamics, impose meanings, or accelerate processing beyond the client’s/patient’s window of tolerance.

### 7.2. Scope and Boundaries of the ETC-AP

The ETC-AP is designed to support the structured observation and transparent interpretation of expressive processes within an ETC framework. The use of numerical scaling in the ETC-AP should not be interpreted as measurement in the psychometric sense. Instead, the numerical profile serves as a visual summary of observed criteria frequencies. It is not a diagnostic instrument and does not assess symptom severity, psychopathology, or trauma exposure. It does not provide normative comparisons, cut-off values, classification, or outcome measurement, and its numerical outputs should not be interpreted as interval- or ratio-scale measurements. Instead, the ETC-AP generates context-dependent profile criteria intended to support within-case formulation, supervision, and interdisciplinary communication. Accordingly, ETC-AP interpretations remain hypothesis-generating and must be integrated with interview material, clinical history, and professional judgment—particularly in trauma-informed contexts where reduced access to specific channels may reflect protective regulation rather than deficits.

### 7.3. The Meaning of “Zero”: Unobserved Criteria Under Current Conditions

A critical interpretive feature of the ETC-AP is its treatment of low or zero values as indicators of unobserved criteria or reduced availability under current conditions, rather than evidence of absence or deficit. Artistic engagement is shaped by situational safety, relational attunement, arousal level, and task context. In addition, the current version of the ETC-AP includes ranges that remain under-defined with respect to formally specified criteria. Accordingly, zero values must be interpreted in light of operational coverage: where criteria are defined, zero indicates that no criteria were observed under the specified criteria; where criteria are not yet defined, zero reflects the current boundary of formalized observation rather than a definitive statement about capacity.

### 7.4. Procedural Uniformity

The ETC-AP is supported by a defined assessment procedure specifying materials, timing, and task sequence. “Uniformity” in this context refers to procedural consistency rather than psychometric standardization. The intent is to increase the clarity and repeatability of the assessment encounter while preserving core art therapy values: non-directiveness, client choice, and process sensitivity (Betts, 2005; Martinsone, 2011). In trauma-informed practice, procedural clarity can itself support safety by reducing uncertainty and enabling informed consent at each step (Butler et al., 2011). At the same time, the approach avoids converting artistic engagement into performance evaluation: the artwork is not treated as a scoreable artifact in isolation, but as part of a regulatory process occurring within a relational and contextual frame.

### 7.5. Practical Implications: Communication, Supervision, and Interdisciplinary Dialogue

A practical benefit of making interpretive logic explicit is improved professional communication. In multidisciplinary services, art therapy observations often need to be conveyed to colleagues who do not share the discipline’s tacit interpretive language. A structured, regulation-oriented ETC-based approach can support clearer dialogue in case formulation, supervision, and documentation by providing shared terms for describing activation, inhibition, and integration patterns. It may also support training by making the reasoning process teachable rather than relying on implicit transmission.

### 7.6. Trauma-Informed Relevance: Regulation, Safety, and Non-Pathologizing Interpretation

The trauma-informed relevance of the ETC-AP is its regulation-oriented lens: assessment observations are organized around safety, arousal, and the client’s/patient’s moment-to-moment capacity for engagement. Trauma-informed practice recognizes that observable behavior and expressive choices may reflect protective strategies shaped by perceived threat and contextual safety (Huo et al., 2023; Butler et al., 2011). Accordingly, reduced access to particular expressive channels is not interpreted as a deficit by default. By differentiating activation, inhibition, and regulatory unavailability, the ETC-AP supports a non-pathologizing stance in which inhibitory patterns may be understood as protective regulation rather than failure, resistance, or a lack of capacity. This constraint is clinically and ethically important in art therapy assessment, where interpretive authority should remain hypothesis-based, context-sensitive, and responsive to consent and pacing.

### 7.7. Applicability Across Client Groups and Contexts

Although this article emphasizes a trauma-informed articulation of the ETC-AP, the operational logic is not inherently limited to trauma-specific populations or settings. The approach is grounded in core ETC principles and organizes observation around general regulatory phenomena—activation, inhibition, and integration—that are relevant across clinical, educational, and rehabilitation contexts. Because it does not rely on symptom measurement, diagnostic categorization, or population-specific norms, it may be applied flexibly across different age groups and levels of cognitive or verbal functioning. Importantly, broader applicability refers to the transferability of the operational logic, not to claims of universal validity or equivalence of meaning across populations.

### 7.8. Limitations and Future Directions

The current developmental stage of the ETC-AP involves several boundaries and methodological constraints that must be explicitly stated to ensure appropriate interpretation and use of the framework. These limitations can be organized into the following five clusters:

- Conceptual and developmental limitations. This article presents a methods-oriented framework at an early developmental stage rather than a completed validation study. While grounded in established ETC theory, the protocol requires further refinement to ensure that its operational definitions fully capture the complexity of artistic expression.
- Lack of psychometric validation. The ETC-AP does not currently report empirical evidence of reliability, validity, or sensitivity to change. The numerical profile outputs are normalized indicators derived from a defined procedure and should not be interpreted as psychometrically calibrated measurements with demonstrated interval-scale properties.
- No direct diagnostic or stand-alone use. The ETC-AP is not a diagnostic instrument and does not provide symptom measurement, clinical classification, or outcome evaluation. It should not be used as a stand-alone basis for case formulation or clinical decision-making. Furthermore, while it utilizes trauma-informed language, the approach does not identify trauma exposure or severity; any trauma-related relevance must remain contextual, clinically situated, and integrated with broader assessment data rather than inferred from the profile alone.
- Scoring and temporal limitations. The current scoring procedure summarizes the relative density of observed criteria across tasks but does not capture the temporal sequencing, duration, or moment-to-moment fluctuation of expressive activity. Consequently, intensity scores reflect the cumulative presence of specific expressive markers within the session rather than time investment, temporal dominance, or the chronological unfolding of the art-making process.
- Restricted generalizability. The illustrative case vignette demonstrates the application of the framework within a specific context but cannot support generalizable claims regarding the instrument’s performance across diverse populations or settings.

Future development priorities include improving operational coverage in currently under-defined inhibition ranges and strengthening process-sensitive documentation through structured observation formats and calibrated exemplars. Programmatic studies focusing on feasibility, inter-rater consistency, and convergent validity are essential to evaluate the framework’s reliability and trustworthiness across clinical and research settings. In summary, the ETC-AP is intended to externalize clinical reasoning and support professional dialogue while remaining subordinate to professional judgment and the client’s/patient’s safety.

## 8. Conclusions

This article presents the current developmental stage of the ETC-AP—a trauma-informed, regulation-oriented approach that makes ETC-based assessment reasoning explicit. By combining a defined three-task administration procedure, criteria-guided observation, and a structured five-step interpretation sequence, the ETC-AP supports the transparent within-case description of activation-inhibition patterns and their organization around integration midpoints. The illustrative case example demonstrates how the approach can generate cautious, context-sensitive hypotheses and support regulation-oriented clinical reflection without providing diagnoses, symptom measurement, or prescriptive treatment plans.

In relation to the research questions, the findings indicate that the descriptive principles of the ETC can be systematically translated into a structured, criteria-guided observation protocol. Furthermore, the use of cumulative scoring combined with normalization procedures (e.g., POMP transformation and proportional weighting) enables qualitative observations to be represented as a coherent visual profile. Finally, the five-step interpretation algorithm provides a transparent and replicable sequence for organizing observations into trauma-informed, regulation-oriented clinical hypotheses.

At the same time, the ETC-AP remains a developmental framework. Its outputs should be understood as normalized profile indicators that summarize observed criteria within the current assessment context. Interpretation requires integration with process observations, interview material, clinical history, and professional judgment, and must remain responsive to safety, consent, and the client’s/patient’s needs. Continued work is needed to refine under-defined inhibition ranges, strengthen training supports and exemplars, and evaluate feasibility and inter-rater consistency across populations and practice settings.

In summary, the ETC-AP provides a structured and communicable framework for translating ETC-informed observation into explicit, criteria-guided clinical reasoning. Its primary contribution is methodological rather than diagnostic: it externalizes interpretive processes that have traditionally remained implicit in art therapy practice. By doing so, it supports transparency, professional dialogue, supervision, and a foundation for cumulative empirical development.

## Data Availability

The original contributions presented in this study are included in the article/Supplementary Material. Further inquiries can be directed to the corresponding author.

## Note

1 Given the fact that Feature 14 is scored jointly across all three produced artworks, the value in row 13 under P+ (column B) is recorded as (5 × 3) + 1.

## Supplementary Materials

The following supporting information can be downloaded at: https://www.mdpi.com/article/10.3390/bs16050640/s1, Supplementary Material S1—Excel-based scoring file.

## Abbreviations

The following abbreviations are used in this manuscript:

ETC	Expressive Therapies Continuum
RSU	Rīga Stradiņš University
ETC-AP	Expressive Therapies Continuum—Assessment Profile

## Appendix A

### Appendix A.1

**Table A1:** Operational Definitions and Activation Ranges of the Refined Expressive Therapies Continuum (ETC) Model.

Description & Clinical Interpretation	Term & Activation Range	Abbreviation
Foundational level: focus on pre-verbal, sensorimotor action and physical engagement with art media.	Kinesthetic-Sensory Level	K-S level
The dimension of physical energy, rhythm, and motor action in the creative process.	Kinesthetic Continuum	K axis
Kinesthetic Over-activation: Erratic, uncontrolled, or destructive motor energy; loss of boundaries.	Extreme Hyper-arousal	Kx+
Constructive Kinesthetic: Intentional, rhythmic movement used to release or channel energy.	Moderate Dominance	K+
K-S Synthesis: Balanced state; movement is purposefully guided by sensory feedback.	Functional Integration	K/S
Kinesthetic Hypo-activity: Low physical energy; passive or hesitant interaction with materials.	Moderate Inhibition	K-
Kinesthetic Inhibition: Total motor paralysis; inability to initiate or sustain physical art-making.	Extreme Hypo-arousal (Block)	Kx–
The dimension of internal physiological sensations and tactile/visual feedback.	Sensory Continuum	S axis
Sensory Overload: Flooded by sensations; “stuck” in textures, smells, or overwhelming visual stimuli.	Extreme Hyper-arousal	Sx+
Sensory Awareness: Deep, focused engagement with the tactile and expressive qualities of media.	Moderate Dominance	S+
S-K Synthesis: Sensory input is successfully processed and expressed through motor action.	Functional Integration	S/K
Sensory Diminishment: Lack of awareness regarding the physical/aesthetic properties of the media.	Moderate Inhibition	S–
Sensory Numbing: Complete disconnection from sensory input; internal or external tactile anesthesia.	Extreme Hypo-arousal (Block)	Sx–
The level focusing on structural organization of imagery (form) vs. Emotional expression.	Perceptual-Affective Level	P-A level
The dimension of form, boundaries, spatial relationships, and literal representation.	Perceptual Continuum	P axis
Perceptual Rigidity: Excessive over-control; hyper-focus on rules/perfection; stiff, lifeless imagery.	Extreme Hyper-arousal	Px+
Perceptual Clarity: Organized structure; clear boundaries and recognizable, literal forms.	Moderate Dominance	P+
P-A Synthesis: Emotions are successfully contained and communicated through a stable visual structure.	Functional Integration	P/A
Perceptual Weakness: Difficulty defining boundaries; vague, fluid, or disorganized spatial relationships.	Moderate Inhibition	P–
Perceptual Fragmentation: Chaotic imagery; inability to perceive or create a “whole” gestalt.	Extreme Hypo-arousal (Block)	Px–
The dimension of emotional experience and expression, accessed via color and fluid media.	Affective Continuum	A axis
Affective Flooding: Uncontrolled emotional discharge; feelings overwhelm the ability to maintain form.	Extreme Hyper-arousal	Ax+
Affective Expressiveness: Vibrant, intentional use of media to communicate and process emotional states.	Moderate Dominance	A+
A-P Synthesis: Balance of feeling and form; emotional energy is expressed within structural clarity.	Functional Integration	A/P
Affective Constriction: Emotional distance; art appears flat, cold, or overly intellectualized.	Moderate Inhibition	A–
Affective Paralysis: Emotional numbing; complete inability to access, identify, or depict feelings.	Extreme Hypo-arousal (Block)	Ax–
The level of complex thought, analytical logic, and metaphorical representation.	Cognitive-Symbolic Level	Co-Sy level
The dimension of analytical thought, sequential planning, and logical verbalization.	Cognitive Continuum	Co axis
Hyper-rationality: Over-intellectualization; obsessive planning that blocks intuitive creative flow.	Extreme Hyper-arousal	Cox+
Cognitive Functioning: Effective problem-solving, logical sequencing, and analytical self-insight.	Moderate Dominance	Co+
Co-Sy Synthesis: Symbols and metaphors are logically integrated and consciously understood.	Functional Integration	Co/Sy
Cognitive Weakness: Difficulty with planning, following sequences, or thinking abstractly.	Moderate Inhibition	Co–
Cognitive Disorientation: Fragmented thoughts; inability to organize ideas or use logical reasoning.	Extreme Hypo-arousal (Block)	Cox–
The dimension of intuition, personal metaphors, and archetypal meaning.	Symbolic Continuum	Sy axis
Symbolic Immersion: Lost in “magical thinking” or private symbols without a link to shared reality.	Extreme Hyper-arousal	Syx+
Symbolic Integration: Meaningful use of metaphors to represent deep self-experience and insight.	Moderate Dominance	Sy+
Sy-Co Synthesis: Deep symbolic metaphors are successfully translated into conscious verbal meaning.	Functional Integration	Sy/Co
Symbolic Poverty: Art is strictly literal/decorative; lack of metaphorical depth or “inner voice.”	Moderate Inhibition	Sy–
Symbolic Void: Complete inability to access or form metaphors; rejection of symbolic meaning.	Extreme Hypo-arousal (Block)	Syx–
Integrative Flow: A state of “flow” where all ETC levels can be accessed and integrated to achieve self-actualization and healing.	Creative Axis	CR axis

### Appendix A.2

**Table A2:** List of art materials and auxiliary tools.

Art Materials	Auxiliary Tools
Graphite pencils (soft, hard)	Scissors (right-handed, left-handed)
Colored pencils (12 colors)	Brushes:
Felt-tip markers (12 colors)	for gouache (three sizes: thin size 2, medium size 12, wide size 24)
Pastel crayons (12 colors)	for watercolors (three sizes: thin size 2, medium size 6, wide size 12)
Watercolors (12 colors)	Water container
Gouache paints (12 colors)	Paper towels/napkins
Finger paints (6 colors)	Modeling tools for plasticine and clay (at least one sculpting tool)
Clay	Glue stick
Plasticine (6 colors)	PVA glue
Polymer clay (grey)	Eraser
White paper (A4, A3, A2)	Pencil sharpener
Colored paper (A4)	Wire, string, or another tool for separating clay from a larger block (if needed)

## Appendix B

**ETC-AP Feature Rating Table**

1. Artwork reference code ___.___.___ 2. Artwork reference code ___.___.___ 3. Artwork reference code ___.___.___	
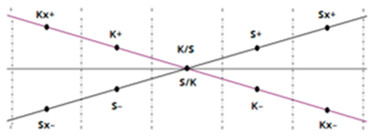

**Kinesthetic (K)-Sensory (S) level**

Feature 1—Intensity of sensory exploration																
1a	Deep absorption in sensory experience													Sx+		
														1	2	3
1b	Engages in sensory exploration										S+					
											1	2	3			
1c	Attempts to avoid contact with art materials	Sx–														
		1	2	3												
Feature 2—Description of kinesthetic activity																
2a	Engages in agitated, restless, or markedly active kinesthetic activity, focusing on the process rather than the final outcome	Kx+														
		1	2	3												
2b	Interrupts or does not complete the initiated activity	Kx+														
		1	2	3												
2c	Destruction of materials	Kx+														
		1	2	3												
2d	Engages in kinesthetic activity, focusing on the process rather than the final outcome				K+											
					1	2	3									
Feature 3—Level of invested energy																
3a	An excessive amount of energy is invested in the creation of the artwork	Kx+														
		1	2	3												
3b	Invests a moderate amount of energy in creating the artwork				K+											
					1	2	3									
3c	The artwork is created using the minimal possible amount of energy													Kx–		
														1	2	3
Feature 4—Rhythm in the art-making process																
4a	Fast or slow rhythmic movements with high intensity aimed at releasing tension	Kx+														
		1	2	3												
4b	Rhythmic movements with moderate intensity				K+											
					1	2	3									
4c	Slow rhythmic movements with a calming quality							K/S								
								1	2	3						
4d	Irregular rhythm of movement													Kx–		
														1	2	3
Feature 5—Range of body movement																
5a	Increased range of body movement	Kx+														
		1	2	3												
5b	Moderate range of body movement				K+											
					1	2	3									
5c	Reduced range of body movement													Kx–		
														1	2	3
Feature 6—Use of space on the page (This indicator is assessed and marked at two levels: K-S and P-A)																
6a	Use of the page: 3/3 and beyond the page margins (more than 80%)	Kx+												Ax+		
		1	2	3										1	2	3
6b	Use of the page: more than 2/3 (70–80%)				K+						A+					
					1	2	3				1	2	3			
6c	Use of the page: 1/3 to 2/3 (30–70%)				K+ P+											
					1	2	3									
6d	Use of the page: reduced or minimal—up to 1/3 (up to 30%)	Ax–												Kx–		
		1	2	3										1	2	3

1. Artwork reference code ___.___.___ 2. Artwork reference code ___.___.___ 3. Artwork reference code *___.___.___*	
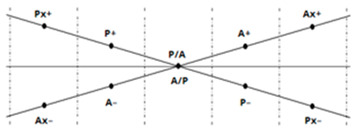

**Perceptual (P)-Affective (A) level**

Feature 7—Properties of form/figure																
7a	Dominance of geometric or highly geometrized forms/figures	Px+														
		1	2	3												
7b	Forms/figures are recognizable, structured, and clear				P+											
					1	2	3									
7c	Forms/figures are recognizable but may be unclear, blurred, or overlapping							P/A								
								1	2	3						
7d	The artwork contains no identifiable forms or figures													Ax+		
														1	2	3
Feature 8—Proportions of forms/figures																
8a	Forms/figures are proportionate to one another				P+											
					1	2	3									
8b	Forms/figures are disproportionate in relation to one another										A+					
											1	2	3			

Feature 9—Size of forms/figures																
9a	Dominance of small-sized forms/figures	Px+														
		1	2	3												
9b	The artwork contains forms/figures of varying sizes and types							P/A								
								1	2	3						
9c	Dominance of large-sized forms/figures													Ax+		
														1	2	3
Feature 10—Use of details																
10a	Excessive focus on details	Px+														
		1	2	3												
10b	Contains a sufficient variety of details without excessive focus on them				P+											
					1	2	3									
10c	Lack of details													Px–		
														1	2	3
Feature 11—Contour lines of forms/figures																
11a	Excessive use of contour lines with a strong focus on them	Px+														
		1	2	3												
11b	Regular, unaccented contour lines				P+											
					1	2	3									
11c	Free and varied use of contour lines and/or contour lines are not used to define or delimit forms/figures							P/A								
								1	2	3						
11d	Dynamically drawn and/or incomplete contour lines										A+					
											1	2	3			
11e	Exaggerated, thick, repeatedly retraced contour lines													Ax+		
														1	2	3
Feature 12—Mixing and blending of colors																
12a	Non-selective color mixing is used													Ax+		
														1	2	3
12b	Color mixing and blending are used										A+					
											1	2	3			
12c	No color mixing or blending is used	Ax–														
		1	2	3												

Feature 13—Color and content congruence										
13a	Color use is incongruent with the content							Ax+		
								1	2	3
13b	Color use is congruent with the content				A+					
					1	2	3			

Feature 14—Repetition of forms/figures (This feature is assessed by considering all three created artworks together)												
14a	Multiple repetition of forms/figures	Px+										
		1	2	3								
14b	Partial repetition of forms/figures				P+							
					1	2	3					
14c	Forms/figures are not repeated							P/A				
								1	2	3		

1. Artwork reference code ___.___.___ 2. Artwork reference code ___.___.___ 3. Artwork reference code ___.___.___	
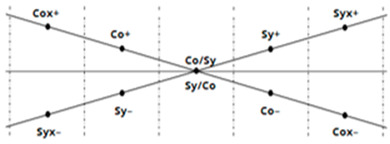

**Cognitive (Co)-Symbolic (Sy) level**
Features 16, 17, and 19 should be assessed with consideration of the post-task inquiry.

Feature 15—Planning and execution of actions												
15a	Is able to plan and sequentially carry out the necessary actions to create the artwork		Co+									
			1	2	3							
15b	Is unable to plan and sequentially carry out the necessary actions to create the artwork									Cox–		
										1	2	3
Feature 16—Integration												
16a	The artwork is conceptually and thematically integrated		Co+									
			1	2	3							
16b	The artwork is not conceptually and thematically integrated									Cox–		
										1	2	3
Feature 17—Words, numbers, schemes, and pictograms depicted in the artwork												
17a	Words, numbers, schemes, and pictograms are used in a logical manner		Co+									
			1	2	3							
17b	Words, numbers, schemes, and pictograms are used in an illogical manner									Cox–		
										1	2	3
Feature 18—Representation of spatial forms/figures and depth												
18a	Forms/figures are represented spatially, and/or the artwork includes perspective		Co+									
			1	2	3							
18b	Forms/figures are not represented spatially, and the artwork does not include perspective						Co–					
							1	2	3			

Feature 19—Meaning of the symbol														
19a	Personal symbol								Sy+					
									1	2	3			
19b	Universal symbol					Co/Sy								
						1	2	3						
19c	Peculiar symbols											Syx+		
												1	2	3
Feature 20—Exploration of the symbol														
20a	Identification with the symbol or excessive use of it											Syx+		
												1	2	3
20b	Ability to relate symbols to personal experiences								Sy+					
									1	2	3			
Feature 21—Metaphor: transfer of meaning based on the comparison of two phenomena														
21a	Use of personal metaphors								Sy+					
									1	2	3			
21b	Use of universal metaphors					Co/Sy								
						1	2	3						
Feature 22—Abstraction														
22a	The abstract artwork has a clear and logical explanation		Co+											
			1	2	3									
22b	The abstract artwork has an unclear and illogical explanation											Cox–		
												1	2	3
Feature 23—Verbal reflection of the client/patient														
23a	The reflection is clear and logical		Co+											
			1	2	3									
23b	The reflection is unclear and/or chaotic											Cox–		
												1	2	3

This Rating Table is intended for the assessment of all three artworks.

When completing the Rating Table, the artwork itself, the art-making process, the verbal reflection of the client/patient, and responses to questions are considered.

Please complete this Rating Table by circling the appropriate number for each criterion:

1 (first artwork), 2 (second artwork), or 3 (third artwork), if the criterion is present in the artwork, observable in the art-making process, or evident in the narrative.

## Appendix C

Kate’s artwork, created during the ETC assessment procedure, with her reflections from the post-task inquiry.

Artwork No. 1

Materials used: A4 paper, crayons

Kate’s reflection: “This is a specific landscape from the surroundings of the hospital. It is a place where we go for walks. We were there today as well. I like this place because it is secluded. I associate it with an inner feeling of loneliness, with being alone. It also reflects how I feel internally at the moment. I like going there because, in summer, water lilies grow there; they don’t at the moment.”

Artwork No. 2

Materials used: A4 paper, crayons

Kate’s reflection: “This is more about feelings. A tree is depicted. I don’t know what kind exactly, it is just some kind of tree. At the bottom, that blackness that has fallen… it is the tree’s own leaves and flowers, already decayed. I wanted a strong, contrasting background. This tree also reflects my inner loneliness. This is how I feel right now.”

Artwork No. 3

Materials used: A4 paper, markers

Kate’s reflection: “I ran out of ideas about what I could do. I wanted to depict one motif in several layers, which is something that simply feels calming and relaxing to me... drawing lines without thinking. But so it’s layered. There isn’t anything deeply complex to think about here. It’s a motif that often appears on wallpaper. I’ve always liked it.”
